# Freeze-Driven Adsorption of Poly-A DNA on Gold Nanoparticles: From a Stable Biointerface to Plasmonic Dimers

**DOI:** 10.1021/acs.langmuir.2c00007

**Published:** 2022-04-11

**Authors:** Yang Ye, Saimei Hou, Xiaomo Wu, Xiaoyu Cheng, Sailing He

**Affiliations:** † National Engineering Centre for Optical Instrumentations, State Key Laboratory of Modern Optical Instrumentation, Centre for Optical and Electromagnetic Research, Zhejiang University, Hangzhou 310058, China; ‡ Ningbo Research Institute, Zhejiang University, Ningbo 315100, China; § Dermatology Hospital of Fuzhou, Xihong Road 243, Fuzhou 350025, China

## Abstract

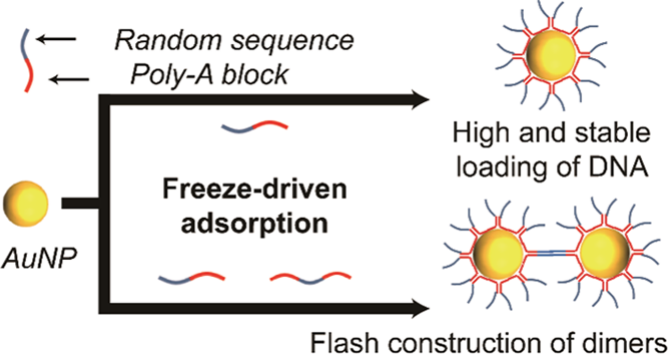

Increasing attention is paid to poly-adenine (poly-A) DNA-functionalized gold nanoparticles due to the high cost of thiols. Freezing is an effective approach for immobilizing poly-A DNA on gold nanoparticles, but its mechanism remains elusive. To cope with this issue, in this paper, some experimental insights are provided. It is shown that (1) the DNA loading density is independent of the length of poly-A. (2) DNA is densely packed on gold nanoparticles, and the biointerface is peculiarly stable, which is not in line with the existing “wrapping” model. (3) Using a DNA-staining dye, thiazole orange, it is shown that poly-A duplex structures are formed on the surface of gold nanoparticles, with evidence given by fluorescence and Raman measurements. An alternative model involving stable poly-A duplexes anchored by finite terminal adenines is proposed. Based on it, a strategy for constructing plasmonic dimers is developed, using freeze-driven adsorption of a DNA sequence with poly-adenine at both ends. This work provides insights into the reaction between poly-A DNA and AuNPs upon freezing and is expected to facilitate related research in biosensor development and nanotechnology.

## Introduction

DNA as a functional polymer has been used in various applications for its unique molecular recognition, programmability, catalysis, and structure-directing functions.^1,2^ Among DNA-functionalized nanomaterials, gold nanoparticles (AuNPs) have attracted wide attention as they can be synthesized easily with high quality and excellent optical properties. Of particular interest are localized surface plasmon resonance (LSPR) effects, which lead to enhanced local fields, such as Raman signals.^3,4^ The good biocompatibility and versatility of DNA-AuNP conjugates have enabled various applications in biosensing, photothermal therapies, drug delivery, and materials science.^5−13^

Thus far, various methods for preparing DNA-AuNP conjugates have been developed.^14^ Conventionally, the conjugation reaction proceeds by reducing charge repulsion via adding a salt (NaCl) slowly, while still maintaining colloidal stability delicately during the process.^15,16^ Liu et al. reported a method of preparing AuNP-DNA conjugates at reduced pH without a salt and attributed its success to the protonation of DNA bases.^17^ Later, the same group reported the freeze-induced conjugation method^18^ and found that DNA was aligned and stretched during a phase change.^19^ While all of the above studies involve thiolated DNA, functional conjugates with nonthiolated DNA are in demand due to the high cost of thiols.^20,21^ Taking advantage of the high affinity of poly-adenine (poly-A), the salt-aging method and low pH method have been applied to poly-A DNA conjugation in several reports.^20,22,23^ The resulting conjugates share similar functions and properties with their thiolated counterpart. A generally accepted adsorption model (the wrapping model) was established, in which DNA wraps around the AuNPs so that all of the adenines in the poly-A block were adsorbed.^20^ A recent study has confirmed the success of conjugating poly-A DNA by freezing.^21^ Although this single-step and reagent-free process is appealing, a few further questions remain to be addressed. First, consecutive adenines have been shown to be essential in the poly-A conjugation process by creating a local hydrophobic environment.^24^ The lateral aligning of DNA upon freezing would lead to the inaccessibility and inactivity of most adenines in the proposed aligned structure, and there seems to be an irreconcilable contradiction. Hence, the interaction between the poly-A block and AuNPs during freezing needs to be studied.

**Scheme 1:**
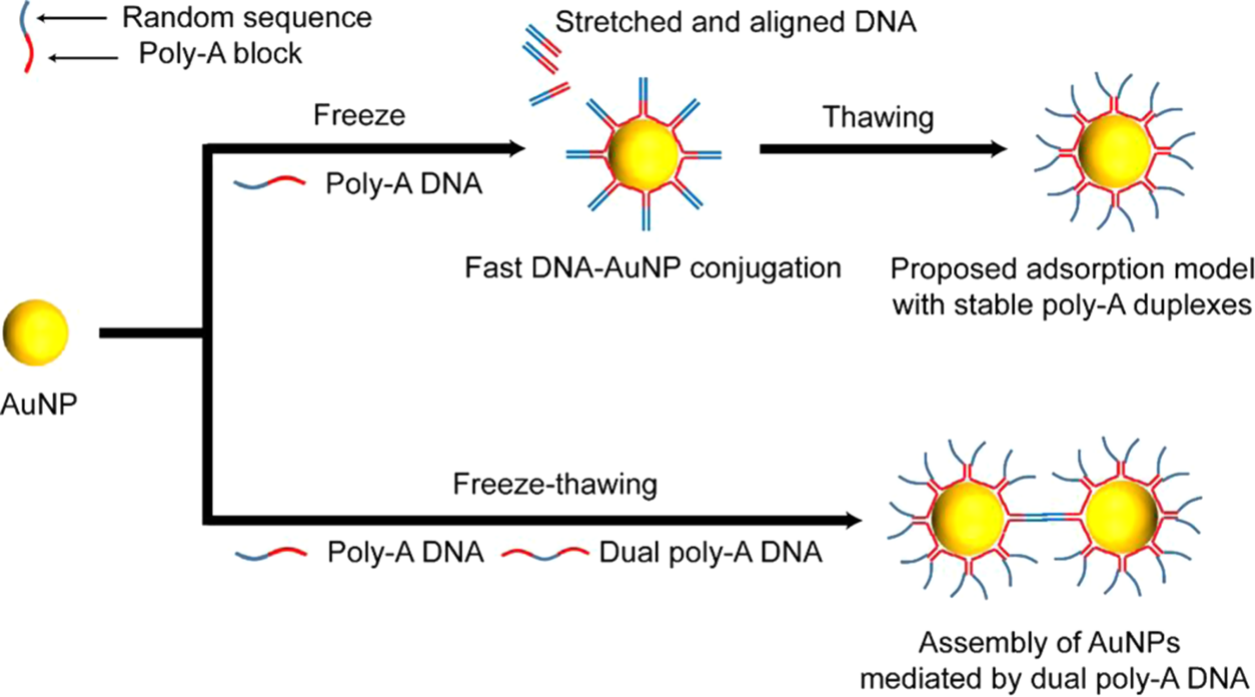
Schematic Diagram Showing the Processes of Freeze-Driven Adsorption of Poly-A DNA on AuNPs Poly-A DNA is stretched and aligned to form poly-A duplexes upon freezing, accompanied by adsorption on AuNPs via finite terminal adenines. The duplex structures are stable on the AuNP surface. When AuNPs are frozen with dual poly-A DNA, a stable assembly of AuNPs can be mediated.

In this paper, mechanistic insights into the freeze-driven poly-A conjugation process are provided. A surprisingly high-density and stable poly-A DNA loading was reported, suggesting a new model of DNA interaction with AuNPs, involving the anchoring of poly-A duplexes (Scheme 1). Additionally, the construction of a AuNP assembly using freeze-driven adsorption of the DNA sequence with poly-adenine at both ends was demonstrated. We anticipate that this work will provide a better understanding of the poly-A DNA conjugation to AuNPs by freezing and facilitate their use in a number of future applications.

## Results and Discussion

**Figure 1:**
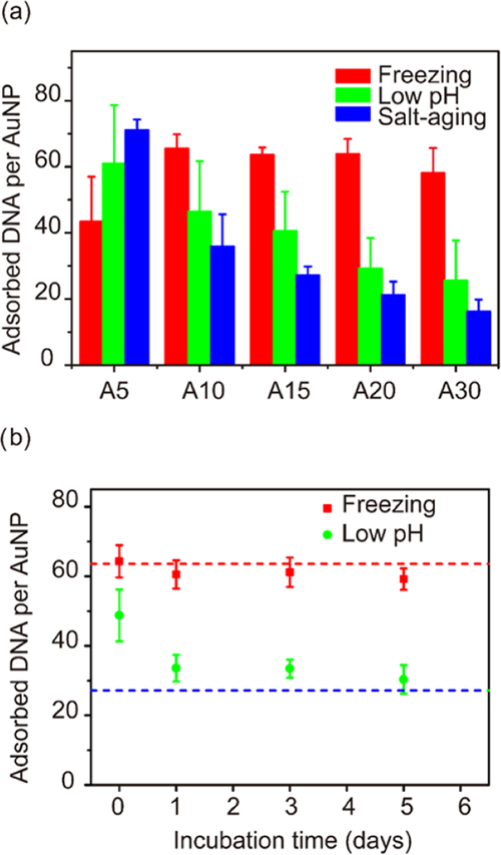
Study of the loading density of poly-A DNA on AuNPs upon freezing. (a) Comparison of the loading density on each AuNP using the freezing, low pH, and salt-aging methods. (b) Change of the loading density as a function of incubation time using the freezing and low pH methods. The red dashed line represents the level of loading density right after a freeze–thaw cycle, and the blue dashed line represents the level of loading density achieved by the salt-aging method.

To understand poly-A adsorption during freezing, the effect of the poly-A length was first investigated. AuNPs (13 nm) were synthesized based on the standard citrate reduction procedure.^25^ Using fluorophore (6-carboxyfluorescein, 6-FAM)-labeled DNA with poly-A ends, we quantified the loading density and compared the freezing results with salt aging and low pH methods (Figure 1a). In the freezing case, the amount of loaded poly-A DNA was quite high, in fact much higher compared with salt aging and low pH methods. Interestingly, the length of poly-A had little impact when the number of adenines was over 10 in the freezing case, in contrast to a significant drop in the loading density in the other two cases. The only exception was the case of a very short poly-A block (A5 DNA), which could be interpreted as a result of labeling failure,^21^ as verified by UV measurements (Figure S2, Supporting Information). The above observations could not be accounted for by the traditional “wrapping model”, which predicted a lower surface density with a longer poly-A block.^20^

The stability of surface-bound DNA was then investigated. Specifically, we compared the loading density of DNA (A15-F and with A15 block) between freezing and low pH methods (Figure 1b). Evidently, the loading density for the low pH method decreased significantly to the level of the salt-aging method on the first day and remained as such for the next 2–5 days, consistent with previous observations.^23^ In the freezing case, however, the loading of DNA was quite stable with slight fluctuations during the long incubation time. The loading of DNA with a longer poly-A block (A20-F and A30-F) using the freezing method exhibited similar stability (Figure S3, Supporting Information). These results indicated that the biointerface formed during the freezing process was peculiarly stable. A different equilibrium state for the freezing method must be considered beyond the wrapping model, as discussed in detail by Liu et al.^26^

Based on the above observations and previous studies,^26,27^ we hypothesized that poly-A DNA interacted with each other to form duplex structures upon freezing. To test it, we used a DNA-staining dye, thiazole orange (TO). TO is a well-established dye that can bind to double strands and duplexes to produce strong fluorescence. After freezing, all DNA sequences with poly-A blocks showed strong fluorescence, ∼4 times higher than samples without freezing (Figures S5 and S6, Supporting Information). Such enhancement was comparable with results obtained at acidic pH reported previously,^26^ indicating a similar, duplex-type structure capable of TO binding. Besides, the fluorescence of non-poly-A sequences under different conditions was almost at the same level. The slightly stronger fluorescence (∼ 30%) of the frozen samples could be attributed to freezing promoted hybridization of very short DNA blocks in the non-poly-A sequences.^28^ These results suggested that poly-A duplexes formed by freezing and produced remarkable fluorescence enhancement, as the fluorescence from hybridization can be excluded.

**Figure 2:**
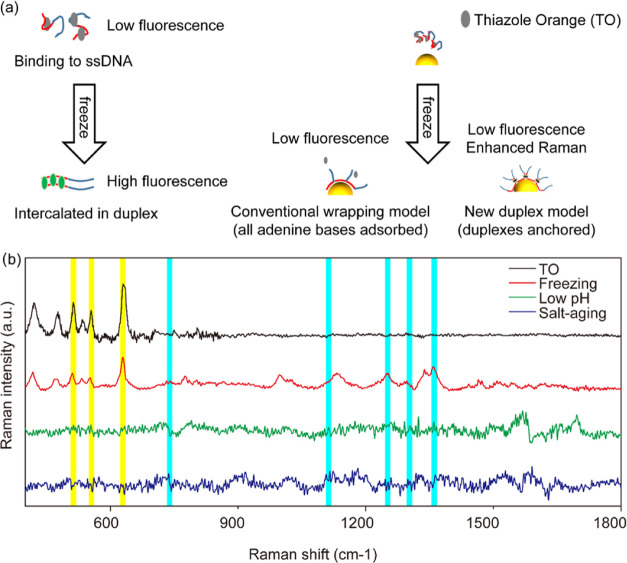
Probing duplex formation by TO staining. (a) Schematic diagram of TO binding to DNA and relevant signal changes: enhanced fluorescence with poly-A duplex, quenched fluorescence, and enhanced Raman with poly-A duplex in the vicinity of gold nanoparticles. (b) Raman spectra of TO-stained DNA-AuNP conjugates prepared by freezing (red), low pH (green), salt-aging (blue), and a concentrated TO solution (black) with the fingerprint peaks of TO (yellow stripes) and adenine (cyan stripes) marked.

Then, the freeze-constructed biointerface involving poly-A duplexes and AuNPs was studied (Figure 2). It is well known that AuNPs exhibit fluorescence quenching ability.^5,29^ That is, the fluorescence of TO is not significant no matter whether TO is free in the solution or near the surface of AuNPs (Figure S4, Supporting Information). On the other hand, the plasmonic properties of AuNPs enable local electromagnetic field enhancement, leading to surface-enhanced Raman scattering (SERS) (Figure 2a).^30^ Therefore, the biointerface was studied using surface-enhanced Raman spectroscopy, including a concentrated TO solution and TO-stained DNA-AuNP conjugates prepared with three different methods (Figure 2b). For concentrated TO solution, three main peaks at 512, 552, and 630 cm^–1^ were observed, of which the assignment can be obtained from the literature.^31,32^ These signature peaks of TO were significant for the freezing sample, while almost none of these bands could be observed for the low pH and salt-aging sample. The strong TO bands in the freezing sample suggested stable poly-A duplexes even after thorough washing, which could not be explained by the wrapping model.

In addition to TO-related Raman bands, adenine-related peaks at 740, 1120, 1177, and 1360 cm^–1^ were expected for all of the DNA-AuNP samples. However, peaks at these positions were almost invisible. In the Raman spectra of a larger scale (Figure S7, Supporting Information), broad bands at about 1350 and 1580 cm^–1^ in all of the DNA-AuNP conjugates indicated an abundance of carbon from DNA strands. These bands confirmed the successful conjugation of DNA by the three methods.^33^ The relatively weak DNA-related characteristic peaks could be rationalized by the small Raman scattering cross sections of oligonucleotides compared with TO.

**Figure 3:**
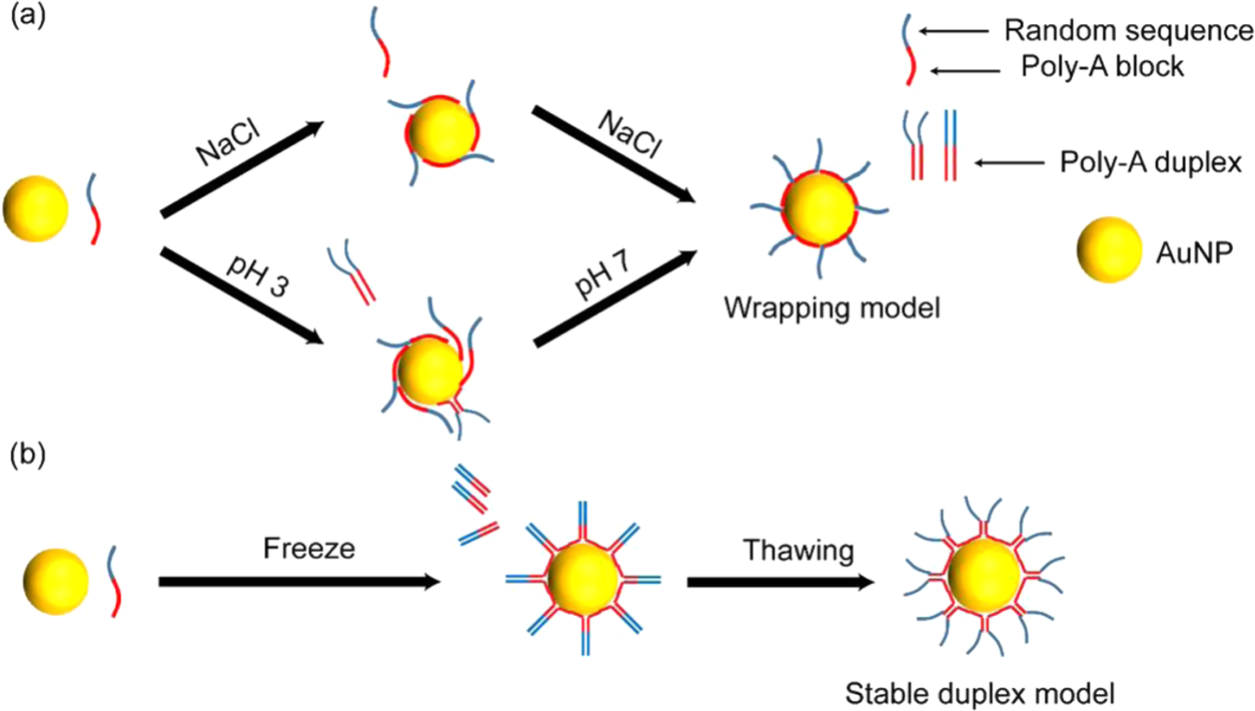
Mechanism of poly-A DNA conjugation to AuNPs. (a) Conventional “wrapping” model. The salt-aging process involves initial DNA adsorption and further adsorption of more DNA with a gradual increase of NaCl concentration. The low pH process involves poly-A duplex formation at acidic pH to facilitate the conjugation process kinetically.^26^ All of the adenines in the poly-A block are adsorbed to achieve thermodynamic equilibrium. (b) Proposed duplex model for the freezing process. Stable poly-A duplexes are anchored by finite terminal adenines, leading to high-density and poly-A length-independent DNA loading.

Given the poly-A length-independent loading, high loading capacity, and stable poly-A duplex of the freezing method, we propose a modified model to describe the freeze-driven adsorption of poly-A DNA on gold (Figure 3). In this model, poly-A DNA is stretched and aligned to form poly-A duplex upon freezing. The coordination interaction between adenine bases and AuNPs is quickly established due to the proximity of the bases to AuNPs by the freeze-concentration effect.^34^ Only a limited number of adenine bases (∼10) can be adsorbed,^21^ and the duplex conformation of the additional adenine bases remains undisturbed in a very short time.

Although limited adsorption of adenines in duplexes was also reported for the low pH process, the initial adsorption of adenines was much less (Figure 1b). As there is room for adsorption, adenine bases will be adsorbed successively, and a wrapping structure will be achieved eventually (Figure 3a). However, the freeze-concentration effect leads to full surface coverage with adenine bases upon freezing (Figure 3b). For the change from duplex to wrapping, not only duplex base pairing but also the interaction between adenine bases and gold have to be overcome. Hence, the freeze-constructed biointerface is stable, accounting for the stability of high-density DNA loading in our work.

As the sticky poly-A block at either end of the sequence can be attached to AuNPs, it is anticipated that dual poly-A (dA) DNA can mediate the assembly of AuNPs. This is of particular interest because the individual plasmon oscillations on different nanoparticles can couple via the near-field interaction to induce unique collective plasmonic properties, such as hot spots in which the EM field is significantly enhanced.^13,30^

**Figure 4:**
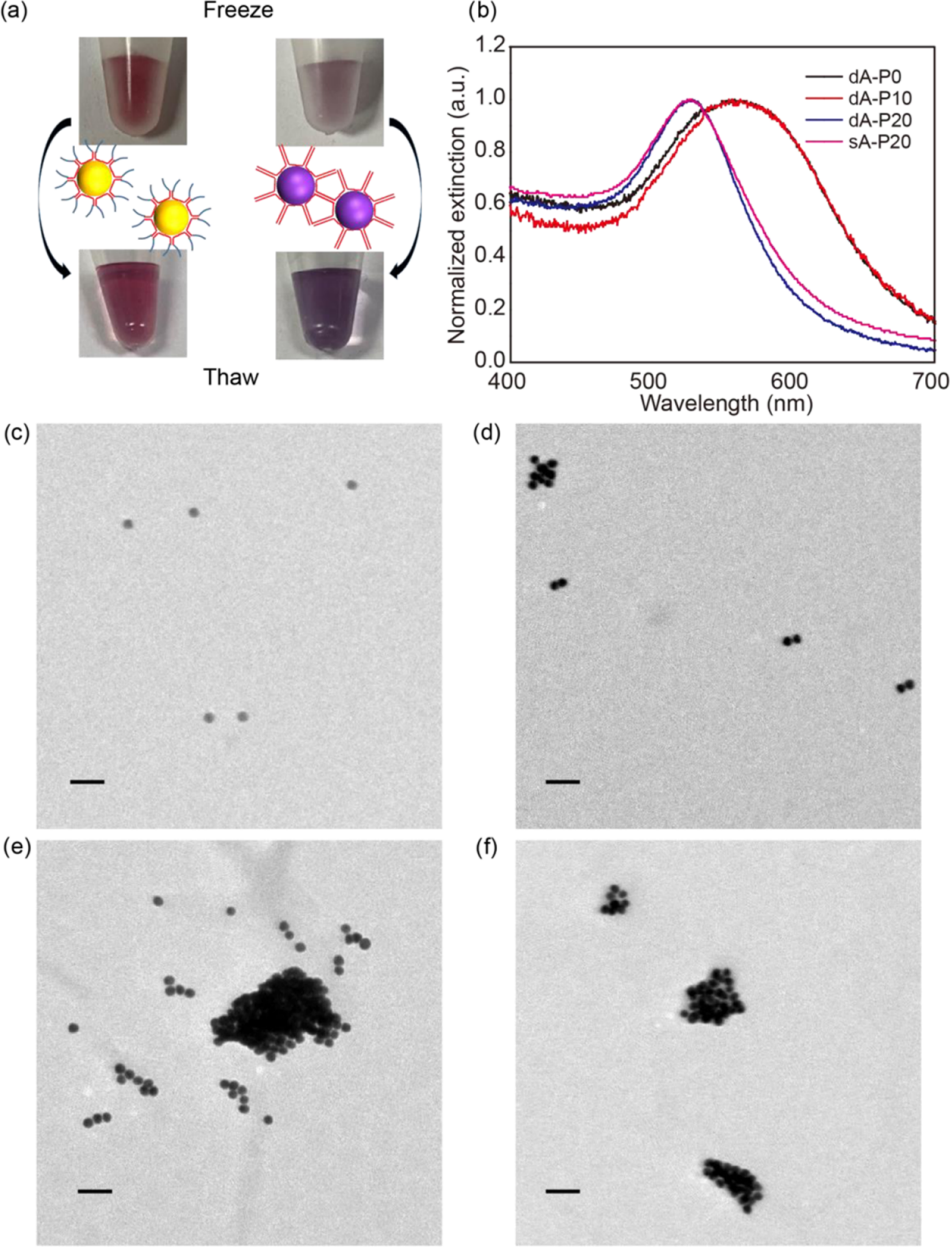
Freeze-constructed assembly of AuNPs using dual poly-A (dA) DNA. (a) Schematic presentation and representative photographs of AuNPs frozen with single poly-A DNA (left) and dual poly-A DNA (right). (b) Normalized UV–vis extinction spectra of DNA-AuNP conjugates prepared with different DNA sequences and representative TEM images of the conjugates obtained with (c) sA-P20, (d) dA-P20, (e) dA-P10, and (f) dA-P0. (Scale bar: 50 nm.)

To test the dA-mediated assembly, four DNAs (dA-P0, dA-P10, dA-P20 with 10 A bases in both ends and a single poly-A (sA) DNA sA-P20 with a 10 A base at the 5′ end, Table 1) were frozen with AuNPs. As expected, the dA-P0 sample changed color to purple after a freeze–thaw cycle, while the red color of the sA-P20 sample was retained. The difference in color indicated successful assembly of AuNPs (Figure 4a), which was further confirmed by UV–vis spectroscopy (Figure 4b). The conjugates prepared with dA-P0 and dA-P10 exhibited a broad peak centered at about 562 nm. The large red shifts of the plasmon band strongly suggested that AuNPs assembled closely to induce significant collective electronic interactions.^35^ The conjugates prepared with dA-P20 have an extinction peak at 530 nm, which was almost identical to the conjugates prepared with sA-P20 rather than the other two dA sequences. We rationalized this phenomenon by the distance-dependent plasmonic properties. The maximum possible length of a 20 base single-strand sequence is ∼13.2 nm according to the literature, which is comparable to the size of AuNPs.^36^ Then, there would be no significant shift in the plasmon band for possible assemblies with such a large interparticle distance due to weak plasmonic coupling.^37^ The existence of the dA-mediated assembly was also evidenced by transmission electron microscopy (Figure 4c–f) and dynamic light scattering (DLS) analysis (Figure S8, Supporting Information). As expected, different levels of assemblies were observed in all of the dA samples, which can be distinguished from irreversible aggregation or large agglomerations induced by drying (Figure S9, Supporting Information).

**Table 1:** Sequences and Modifications of the Oligonucleotides

name	sequences and modifications (from 5′ to 3′)
A5-F	AAAAATTTTTATGATGTTCGTTGTG-FAM
A10-F	AAAAAAAAAATTTTTATGATGTTCGTTGTG-FAM
A15-F	AAAAAAAAAAAAAAATTTTTATGATGTTCGTTGTG-FAM
A20-F	AAAAAAAAAAAAAAAAAAAATTTTTATGATGTTCGTTGTG-FAM
A30-F	AAAAAAAAAAAAAAAAAAAAAAAAAAAAAATTTTTATGATGTTCGTTGTG-FAM
A20	AAAAAAAAAAAAAAAAAAAA
A30	AAAAAAAAAAAAAAAAAAAAAAAAAAAAAA
R20	AATCTGTCAAGCAGCAGCAA
R30	AGAATTTATAAGCACCACAACGAACATCAT
A30-P20	AAAAAAAAAAAAAAAAAAAAAAAAAAAAAATTGCTGCTGCTTGACAGATT
sA-P20	AAAAAAAAAATTGCTGCTGCTTGACAGATT
sA-P10	AAAAAAAAAATTGCTGCTGC
dA-P20	AAAAAAAAAATTGCTGCTGCTTGACAGATTAAAAAAAAAA
dA-P10	AAAAAAAAAATTGCTGCTGCAAAAAAAAAA
dA-P0	AAAAAAAAAAAAAAAAAAAA

**Figure 5:**
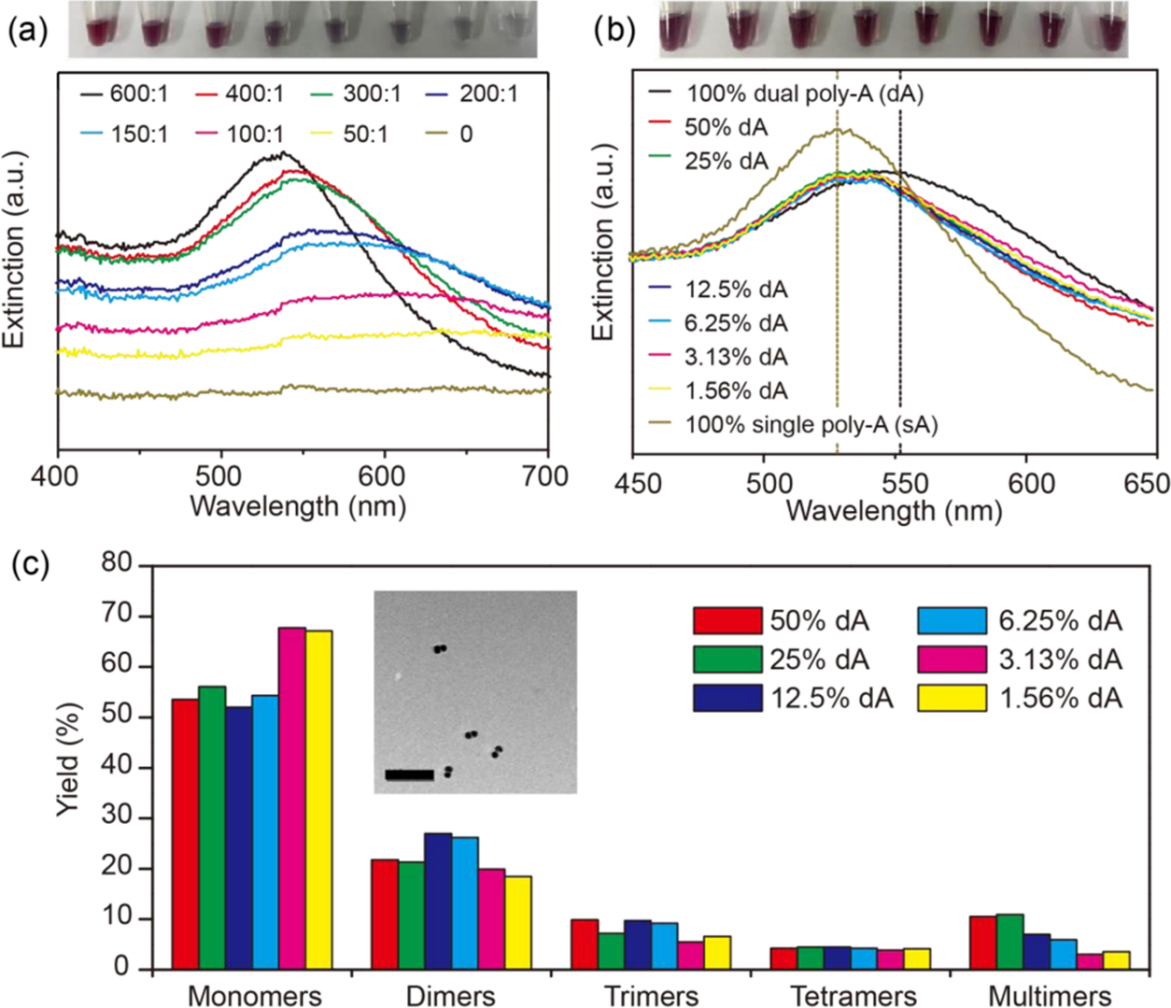
Freeze-induced construction of plasmonic assemblies. (a) Photograph (top) and UV–vis extinction spectra (bottom) of DNA-AuNP conjugates with different DNA/AuNP ratios; the DNA/AuNP ratio decreases from left (600:1) to right (0). (b) Photograph (top) and UV–vis extinction spectra (bottom) of DNA-AuNP conjugates prepared with different concentrations of the dA linker at a constant DNA/AuNP ratio of 400:1 (from left to right: 100% dA, 50% dA, 25% dA, 12.5% dA, 6.25% dA, 3.13% dA, 1.56% dA, 100% sA). The extinction peaks of the 100% dA sample and 100% sA sample are marked with dashed lines in the corresponding color. (c) Statistical distribution of monomers and multimers for the different diluted dA samples. Assemblies composed of more than 10 nanoparticles were ignored as they could be formed during the preparation of TEM grids. (At least 800 nanostructures and 30 images were analyzed for each sample.) Inset: representative TEM image of well-defined dimers prepared with 12.5% dA linkers (scale bar: 100nm).

With the ability to assemble AuNPs, we further investigate the construction of well-defined nanostructures by controlling experimental conditions. Among different kinds of plasmonic nanostructures, the dimer has attracted much attention for the high coupling rate in the gap.^38,39^ It was expected that nanoparticles tend to form smaller assemblies when the number of effective dA linkers decreased. At first, we tried to control the size of assemblies by decreasing the ratio of dA DNA:AuNPs directly (Figure 5a). When the DNA/AuNP ratio was below 300:1, the AuNPs aggregated irreversibly as reflected in blue or gray color. The UV–vis spectra showed that AuNPs tended to aggregate massively when the DNA/AuNP ratio decreased. This result was consistent with a high DNA concentration needed for thiolated DNA freezing,^19^ as a deficiency of DNA will lead to irreversible aggregation. When the dA DNA/AuNP ratio was at 600:1, the color and extinction peak of the DNA-AuNP conjugates were almost identical to those of AuNPs modified by single poly-A DNA. That is, the cross-linking between AuNPs was reduced remarkably with a sufficiently high DNA concentration.

To retain the stability of AuNPs, we maintained the DNA/AuNP ratio at 400:1 but made a serial dilution to the dA DNA (dA-P10) by single poly-A sequences (Figure 5b). The conjugates obtained with different concentrations of dA linkers were characterized by UV–vis spectroscopy. The extinction bands of all of the diluted dA samples fell in between the bands of the pure dA sample (peak at ∼550 nm) and the pure sA sample (peak at ∼ 530 nm), suggesting a considerable plasmonic coupling between particles as well as a degree of control over the size of assemblies. To evaluate the dimer yield, a statistical investigation was performed based on abundant TEM images (Figure S10, Supporting Information). The distribution of assemblies with different coordination numbers was depicted in the histogram (Figure 5c). The overall trend of our results showed a decreasing yield of multimers (assemblies with 5–10 nanoparticles) when the concentration of dA DNA decreased. This was reasonable, as the decrease of the effective dA linkers reduced the possibility of cross-linking. When the dA linkers were diluted to 12.5 or 6.25%, the yield of dimers increased to about 27%. The well-defined dimers prepared with 8-fold diluted dA linkers are shown in the inset of Figure 5c. Further dilution of dA linkers could reduce the yield of dimers instead, along with a dramatic increase of monomers. Overall, the samples prepared with diluted dA-linkers showed a yield higher than 19% before purification, which is reasonable compared to other dimer preparing methods reported in the literature (10% by hydrophobic molecules, 20% by dithiols, and 23% by Cu(I)-catalyzed azide–alkyne cycloaddition).^40,41^ The freeze-induced dimer fabrication by deploying dual poly-A linkers is promising, especially considering the simple reactants, facile reaction conditions, and convenient operation it involves.

## Conclusions

In summary, we systematically studied freeze-driven poly-A DNA adsorption on gold nanoparticles. The loading density of the freezing method is independent of the poly-A length and the densely loaded poly-A DNA is peculiarly stable, which is different from typical solution-phase methods. Based on the identification of poly-A duplex formation, we proposed a new model: poly-A duplexes are formed and anchored by finite terminal adenines upon freezing, and the additional duplex structures are energetically stable in the conjugates. The freeze-concentration effect and the stable conformation adopted by the DNA strands allow for the cross-linking of AuNPs, which is important in the fields of plasmonics and metamaterials. Hence, we develop a novel strategy for the construction of plasmonic nanoparticle assembly without the need for DNA hybridization. This work has provided new insights into the reaction between poly-A DNA and AuNPs upon freezing and will facilitate related research in biosensor development and nanotechnology.

## Experimental Section

### Materials

Hydrogen tetrachloroaurate (III) hydrate (HAuCl_4_·3H_2_O) and 2-mercaptoethanol (2-ME) were purchased from Sigma-Aldrich. Sodium chloride and trisodium citrate dihydrate were purchased from Sinopharm Chemical Reagent Co., Ltd. Phosphate-buffered saline (10 × PBS) and thiazole orange (TO) were obtained from Solarbio Science & Technology Co., Ltd. Deionized (DI) water and oligonucleotides were purchased from Sangon Biotech. The sequences and modifications of the DNAs are shown in Table 1. All chemicals were used as received without further purification, and DI water was applied in all of the procedures in experiments.

### Synthesis of AuNPs

AuNPs (13 nm) were synthesized based on the standard citrate reduction procedure. In brief, 1 mL of 25 mM HAuCl_4_ was added to 89 mL of H_2_O and then heated to boiling. Subsequently, 10 mL of 10 mM trisodium citrate was added quickly with vigorous stirring. The solution was kept boiling and stirred until a color change of the solution from pale yellow to wine red and then cooled to room temperature. The prepared AuNP solution was condensed to ∼10 nM by centrifugation and stored at 4 °C.

### Functionalization of AuNPs with Poly-A DNA

Briefly, 200 μL of AuNP solution was mixed with 8 μL of 100 μM poly-A DNA. In a typical freezing method, the mixture was placed in a freezer (−20 °C) for 2 h, or until frozen. Afterward, the frozen solution was thawed at room temperature. For comparison, functionalization of AuNPs using other methods (e.g., salt aging or low pH) was also carried out. For the salt-aging method, the mixture was incubated overnight. Then, NaCl (1 M) was gradually added up to 300 mM over a time period of 10 h. Afterward, the mixture was incubated overnight again. For the low pH method, 4 μL of citrate buffer (500 mM trisodium citrate, pH 3) was added to the mixture. After 5 min of incubation at room temperature, the pH of the AuNP solution was adjusted back to neutral.

### Quantification of the DNA Loading Density

A well-established fluorescence-based method was used to quantify the loaded DNA on AuNPs.^42^ Briefly, DNA used in these studies was labeled with carboxyfluorescein (FAM) at the end. After loading DNA onto AuNPs and removing free DNA by washing, 2-ME was used to completely displace the surface-bound DNA strands via an exchange reaction. Then, the released DNA was quantified by the fluorescence of the supernatant after centrifugation. To confirm that 2-ME completely displaces poly-A DNA, the precipitates were redispersed in the same buffer with 2-ME and incubated for a second round. The fact that no fluorescence was observed in the second displacement stage indicated that 2-ME completely displaced poly-A DNA in the first incubation.

FAM-labeled DNA (A5-F, A10-F, A15-F, A20-F, and A30-F) was conjugated to AuNPs by three different methods following the protocol described above. The obtained DNA-AuNP conjugates were centrifuged (12 000 rpm, 15 min) to remove the free DNA and washed with 10 mM PBS buffer (0.1M NaCl, pH 7.4) three times. Finally, 2-ME was added (final concentration 20 mM) to each 200 μL of the sample, which was incubated overnight with shaking at room temperature. Released DNA strands were then separated via centrifugation and the fluorescence was measured with a fluorescence microplate reader (SpectraMax M5). The number of DNA loaded on AuNPs was determined with standard curves by adding the same DNA of known concentrations in the same buffer conditions (10 mM PBS, 20mM ME, 0.1 M NaCl, pH 7.4). Concentrations of AuNPs were determined via the extinction of UV–vis spectroscopy.^20^

### Test of Loading Stability

To compare the loading stability of DNA using the freezing method with the low pH method, the DNA density on AuNPs was measured as a function of time. Typically, the modification of A15-F was the same as described above, and the obtained samples were stored at room temperature and pH 7 until sampling. The time points for sampling were 0, 1, 3, and 5 days. At each time point, 50 μL of DNA-AuNP solution was collected from each sample, and the DNA loading density at the time point was determined following the protocol outlined above.

### Staining DNA with TO

The DNA-staining dye TO was added (final concentration 2 μM) to 1 μM DNA samples (A20, A30, R20, R30, and A30-P20). The freezing sample was frozen at −20 °C for 2 h and then thawed at room temperature. The low pH sample was adjusted to pH 3.0 by adding citrate buffer (500 mM trisodium citrate, pH 3) with 5 min of incubation and the control sample was kept without further operation. The fluorescence was measured with a fluorescence spectrophotometer.

### Staining DNA-AuNP Conjugates with TO

DNA A30-P20 was conjugated to AuNPs following the protocol described above. The obtained DNA-AuNP conjugates were centrifuged (12 000 rpm, 15 min) to remove the free DNA and washed with 10 mM PBS buffer (0.1 M NaCl, pH 7.4) twice. After 30 min of incubation with 2 μM TO at room temperature, the samples were centrifuged and redispersed in water for later characterization.

### Raman Measurements of TO

The samples were dripped onto a silicon wafer coated with a thin layer of gold. The 50 nm gold layer was prepared by electron beam evaporation. The thin layer of gold was used to further boost SERS signals. The sessile droplet was kept stationary overnight to evaporate the water. Then, the SERS spectra were recorded on a LabRAM HR Evolution confocal microprobe Raman system (Jobin-Yvon). The excitation line was 532 nm, and the integration time was 15 s. Baselines with different numbers of points were used to correct background fluorescence or highlight the fingerprint peaks.

### Preparation of Assemblies Mediated by DNA with Adenine in Both Ends (dA DNA)

Dual-A10 sequences (dA-P0, dA-P10, dA-P20) were conjugated to AuNPs by the freezing method. After a freeze/thaw cycle, the samples were centrifuged and redispersed in water for later characterization and use. To further control the yield of dimers, 200 μL of AuNP solution was mixed with dA-P10 of varying concentrations (6, 4, 3, 2, 1.5, 1, 0.5, 0 μM) or dA-P10 diluted with sA-P10 (2, 1, 0.5, 0.25, 0.125, 0.0625 μM dA-P10, while the total DNA concentration was fixed at 4 μM). After a freeze/thaw cycle, the samples were centrifuged and redispersed in water for later characterization.

### Characterization

UV–vis absorption spectra were obtained with a Shimadzu 2550 UV–vis scanning spectrophotometer at room temperature. Dynamic light scattering measurements were conducted using a Zetasizer Nano ZS-90 (Malvern) at room temperature. The structures of nanoparticles and assemblies were taken with a JEOL JEM-1200X transmission electron microscope operated at 80 kV in bright-field mode. Transmission electron microscopy (TEM) samples were prepared by dropping 5 μL of the purified sample solution on a carbon-coated grid. The grid was kept at room temperature for 24 h to allow drying.

## Supporting Information Available

UV–vis spectra, additional loading stability test, fluorescence measurements, supplementary Raman spectra, DLS characterization, and TEM characterization (PDF)
