# Supplementary material for: Freeze-Driven Adsorption of Poly-A DNA on Gold Nanoparticles: From a Stable Biointerface to Plasmonic Dimers
Source: Langmuir. 2022 Apr 11;38(15):4625–32. doi: 10.1021/acs.langmuir.2c00007 (PMC9022424; doi:10.1021/acs.langmuir.2c00007)
Supplement: Supplementary file 1 — la2c00007_si_001.pdf [file la2c00007_si_001.pdf]

Supporting Information

Freeze-Driven Adsorption of poly-A DNA on Gold Nanoparticles: from Stable Biointerface to Plasmonic Dimers

Yang Ye,[†,‡] *Saimei Hou,*[†] *Xiaomo Wu,*[§] *Xiaoyu Cheng,*[*,†,‡] *and Sailing He*[*,†,‡]

†National Engineering Centre for Optical Instrumentations, State Key Laboratory of Modern Optical Instrumentation, Centre for Optical and Electromagnetic Research, Zhejiang University, Hangzhou, 310058, China

‡Ningbo Research Institute, Zhejiang University, Ningbo, 315100, China

§ Dermatology Hospital of Fuzhou, Xihong Road 243, Fuzhou, 350025, China

Content

1. Characterization of newly synthesized AuNPs 2
2. UV-vis spectra of AuNPs frozen with different length of poly-A 3
3. Loading stability of A20-F and A30-F by freezing 4
4. Fluorescence quenching of dyes with AuNPs 5
5. Fluorescence intensity of TO-stained DNA with different sequence 6
6. Fluorescence intensity of TO-stained DNA before and after freezing. 7
7. Large-scale Raman spectra of TO stained DNA-AuNP conjugates 8
8. Dynamic light scattering measurement for hydrodynamic diameters of DNA-AuNPs conjugates. 9
9. TEM images of AuNPs showing drying-induced agglomeration 10

10. TEM images of DNA-AuNP conjugates prepared with different concentration of dA linker at a constant DNA/AuNP ratio 11

1. Characterization of newly synthesized AuNPs

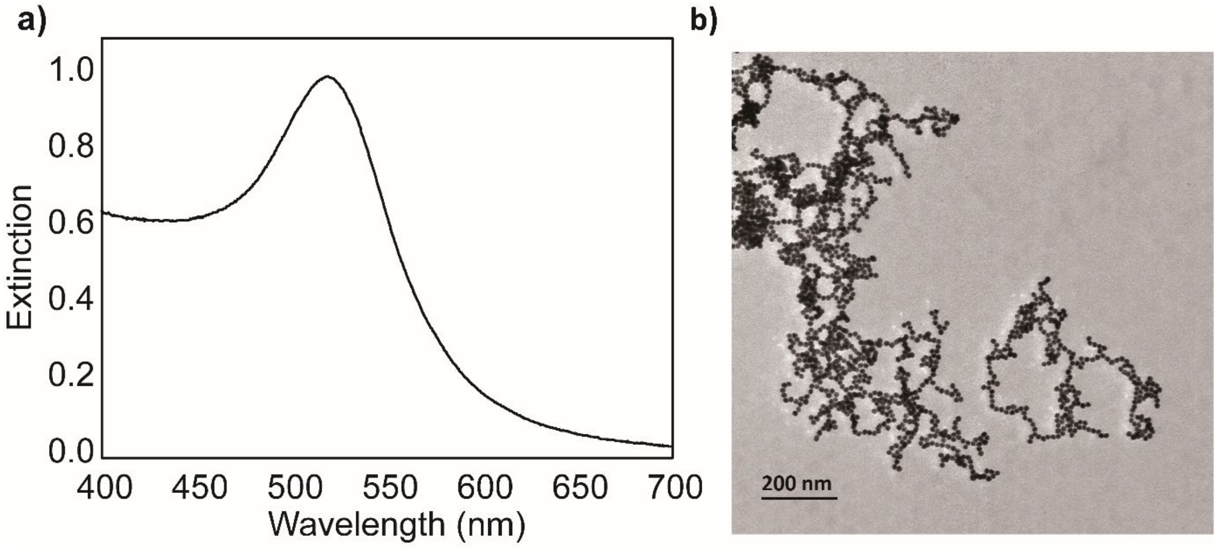

Figure S1. Characterization of newly synthesized AuNPs. (a) UV-Vis extinction spectrum and (b) representative TEM images of the nanoparticles synthesized. The absorption peak is at 518 nm and the diameter of the nanoparticles is about 13nm.

2. UV-vis spectra of AuNPs frozen with different length of poly-A

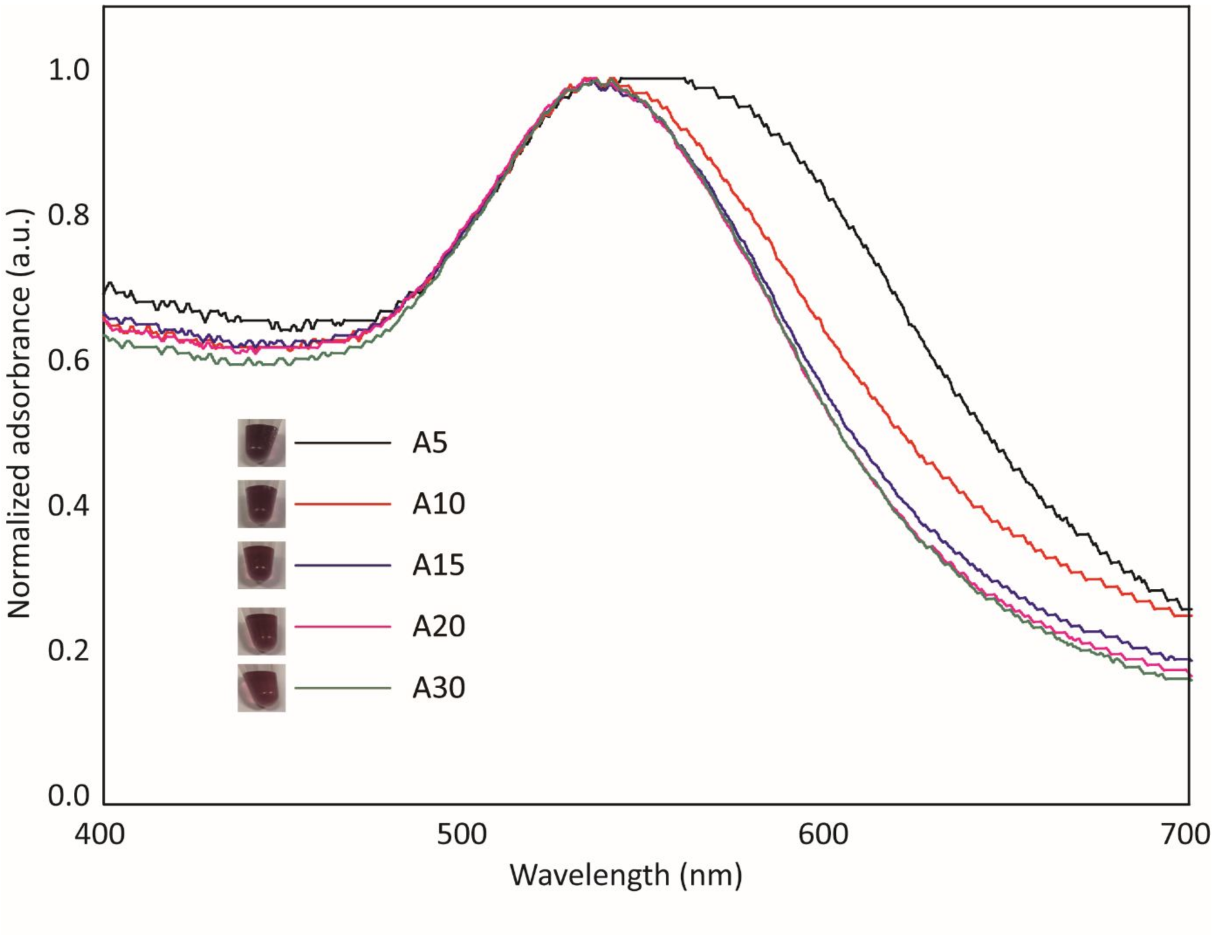

Figure S2. Normalized UV−Vis extinction spectra and corresponding photographs of AuNPs frozen with A5-F, A10-F, A15-F, A20-F, A30-F. The purple color of the solution after freeze-thawing and the siginificant blue-shift of the peak indicate irreversible aggregation of the AuNPs, that is, A5-F fail to conjugate to AuNPs to proctect the particles from aggregation.

3. Loading stability of A20-F and A30-F by freezing

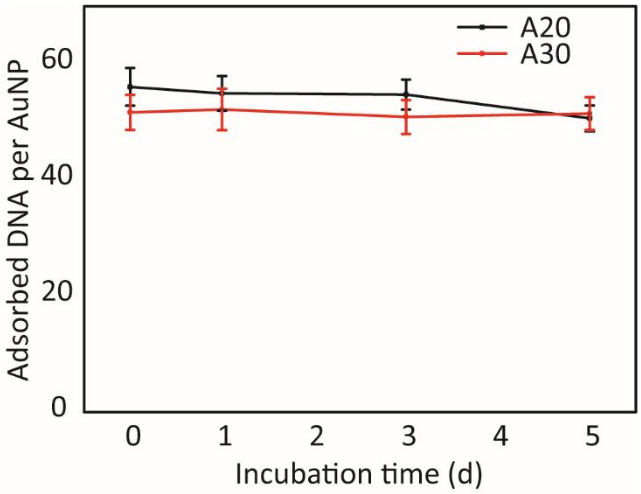

Figure S3. Change of DNA loading density (A20-F, A30-F) as a function of incubation time using the freezing method.

4. Fluorescence quenching of dyes with AuNPs

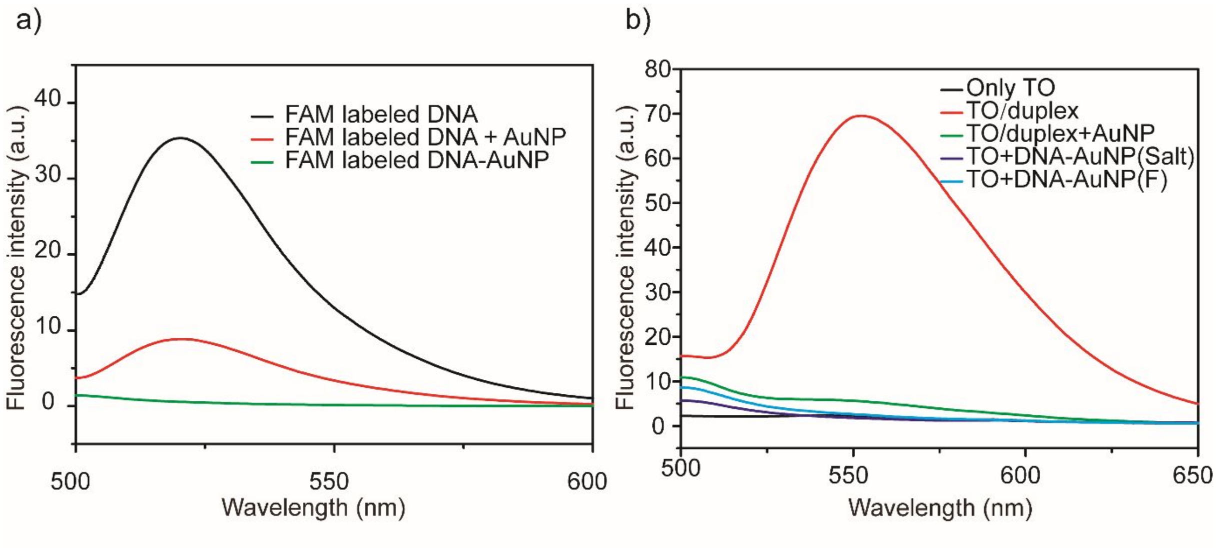

Figure S4. Fluorescence quenching of dyes with AuNPs. (a) Fluorescence spectra of FAM labeled DNA, FAM labeled DNA mixed with AuNPs and FAM labeled DNA adsorbed on the surface of AuNPs. The significant decrease of the fluorescence intensity suggests drastic quenching with AuNPs. (b) Fluorescence spectra of TO, TO stained duplex and TO with gold nanoparticles. When TO is mixed with the duplex structure, strong fluorescence emission at about 550 nm is observed. When TO is mixed with AuNPs or DNA-AuNPs conjugates, almost no fluorescence signals are observed, no matter whether the conjugates are prepared by salt-aging or freezing.

5. Fluorescence intensity of TO-stained DNA with different sequence

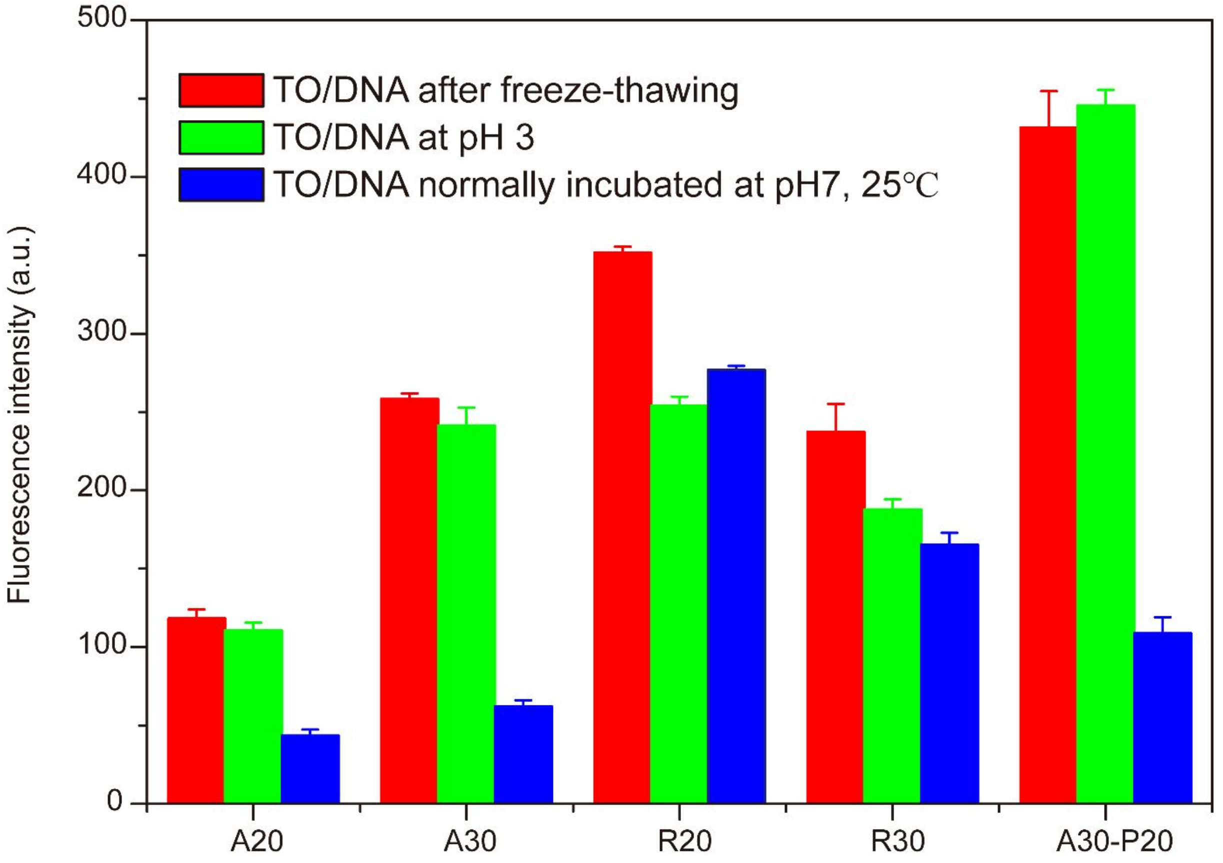

Figure S5. Fluorescence intensity of TO-stained DNA with different sequence.

6. Fluorescence intensity of TO-stained DNA before and after freezing.

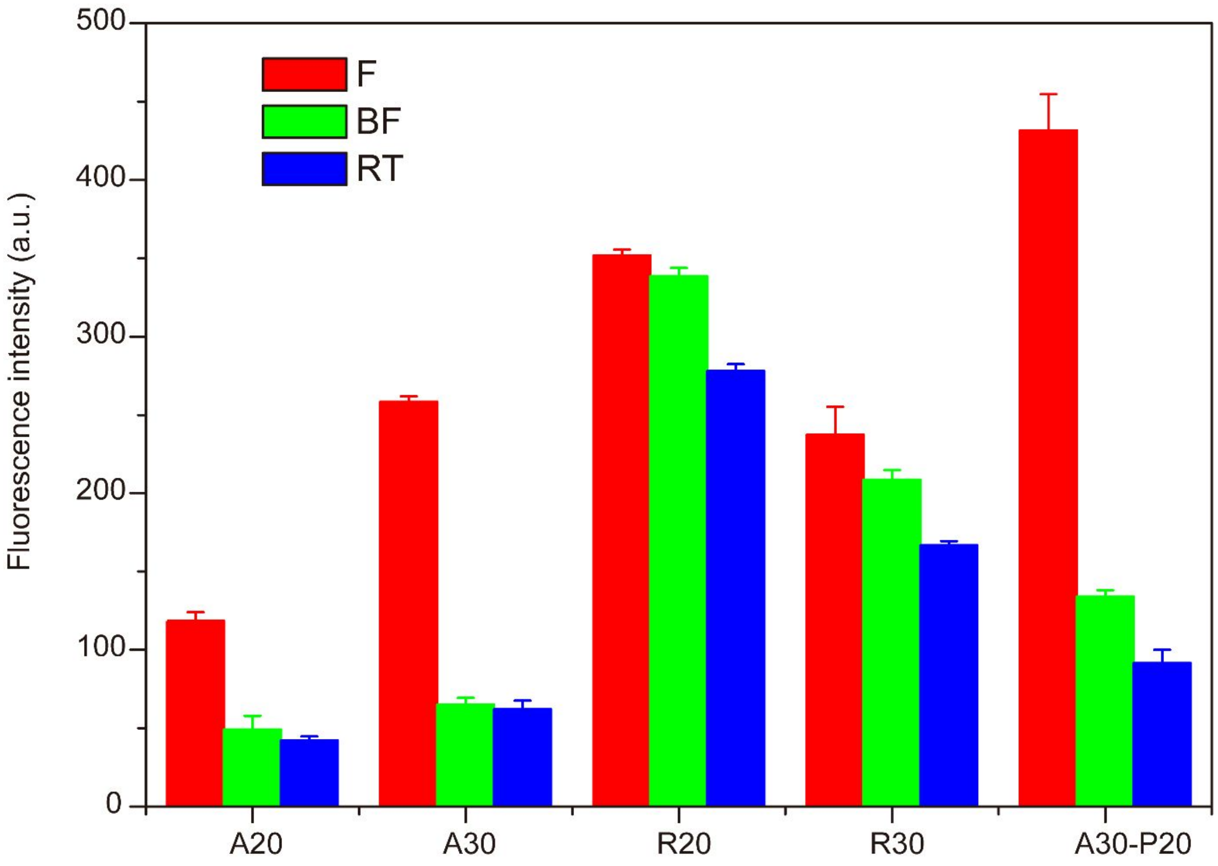

Figure S6. Fluorescence intensity of TO-stained DNA before and after freezing. F – DNA treated by freeze-thawing and balanced to room temperature, BF – DNA cooled at -20 ^{o}C for 10min before freezing, RT – DNA incubated at room temperature. The huge fluorescence enhancement with freezing samples of poly-A sequences indicates that a unique poly-A structure is formed by freezing.

7. Large-scale Raman spectra of TO stained DNA-AuNP conjugates

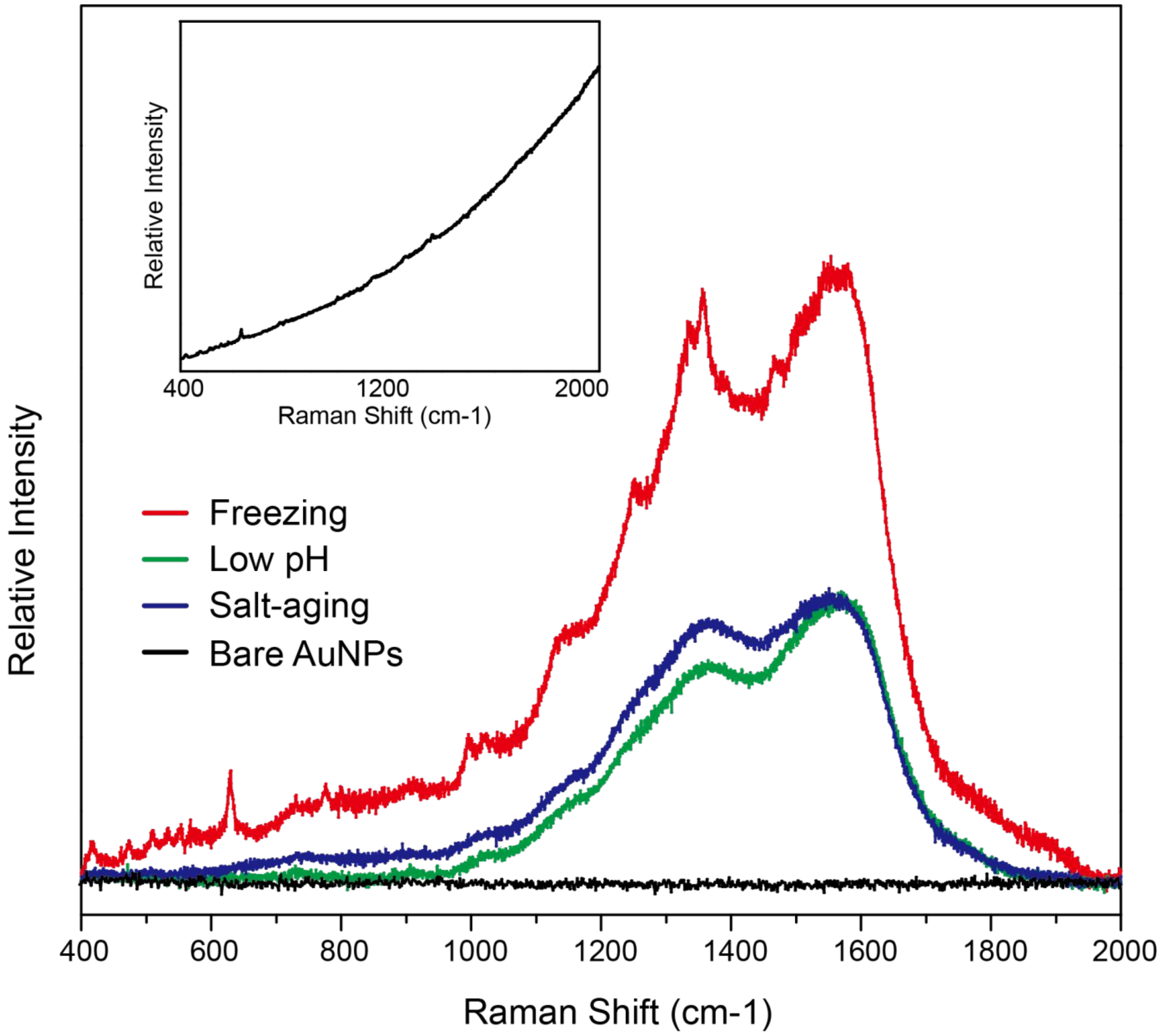

Figure S7. Large-scale Raman spectra of TO stained DNA-AuNP conjugates prepared by freezing (red), low pH (green) and salt-aging (blue), the Raman spectra of bare AuNPs without DNA is represented as a control. TO related peaks are significant only in the freezing sample. Broad bands at about 1350 and 1580 cm^{-1} for all the samples could be assigned to carbon bands of DNA.[1, 2] The inset shows the raw spectrum of pure TO, indicating a sloping background of fluorescence which needs to be corrected.

8. Dynamic light scattering measurement for hydrodynamic diameters of DNA-AuNPs conjugates.

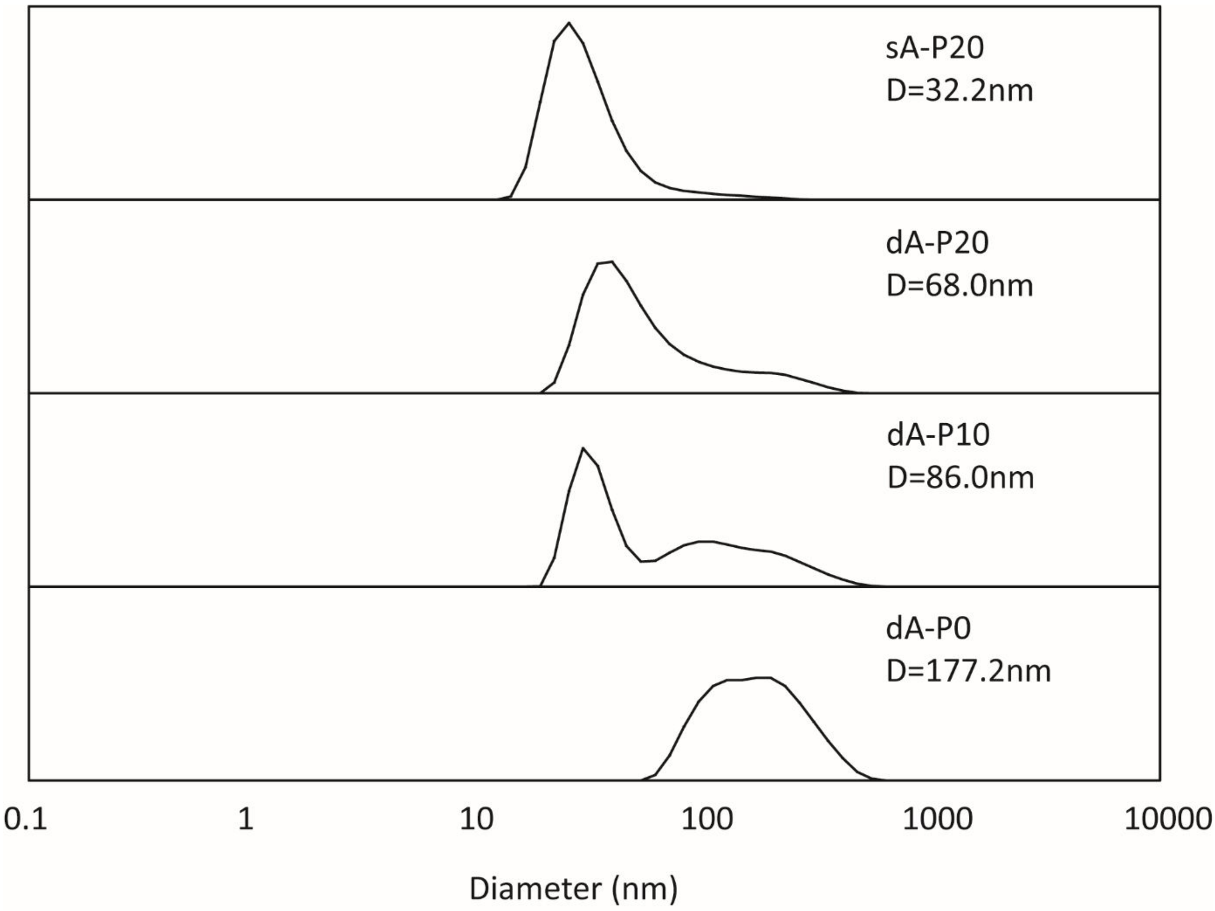

Figure S8. Dynamic light scattering measurement for hydrodynamic diameters of the conjugates or the assemblies obtained with sA-P20, dA-P20, dA-P10 and dA-P0. The emergence of multiple peaks provides strong evidence that dual poly-A (dA) DNA can mediate the crosslink and assembly of AuNPs.

9. TEM images of AuNPs showing drying-induced agglomeration

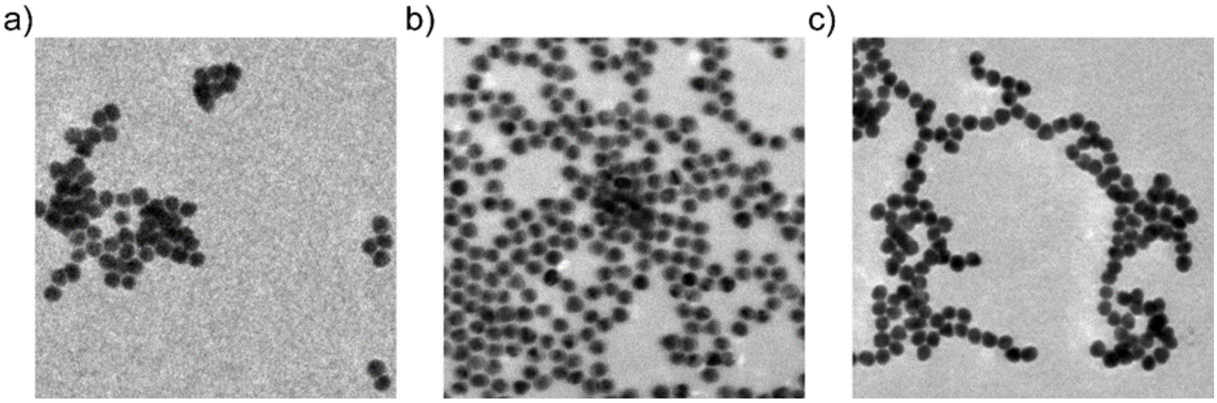

Figure S9. Representitive TEM images of (a) dA-P10 modified AuNP, (b) sA-P10 modified AuNP and (c) newly synthesized AuNP showing clusters with tens of particles in all the samples. The drying-induced agglomerations are always extensive and consecutive. Since such clusters could form by both poly-A-mediated assembly and drying-induced agglomerations, miscounting of those clusters is fatal to evaluating the actual yield of nanostructures.

10. TEM images of DNA-AuNP conjugates prepared with different concentration of dA linker at a constant DNA/AuNP ratio

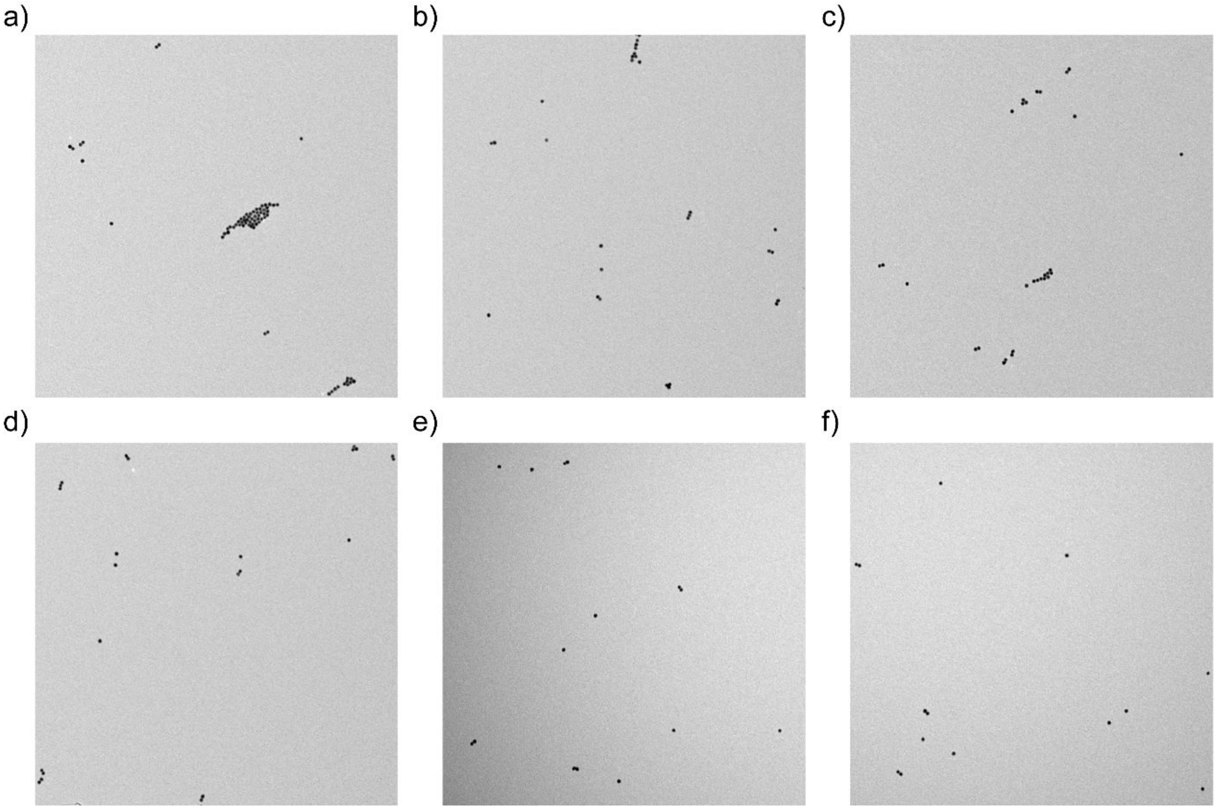

Figure S10. Representative TEM images of DNA-AuNP conjugates prepared with (a)50% dA-P10, (b) 25% dA-P10, (c)12.5% dA-P10, (d) 6.25% dA-P10, (e) 3.13% dA-P10 and (f) 1.56% dA-P10.

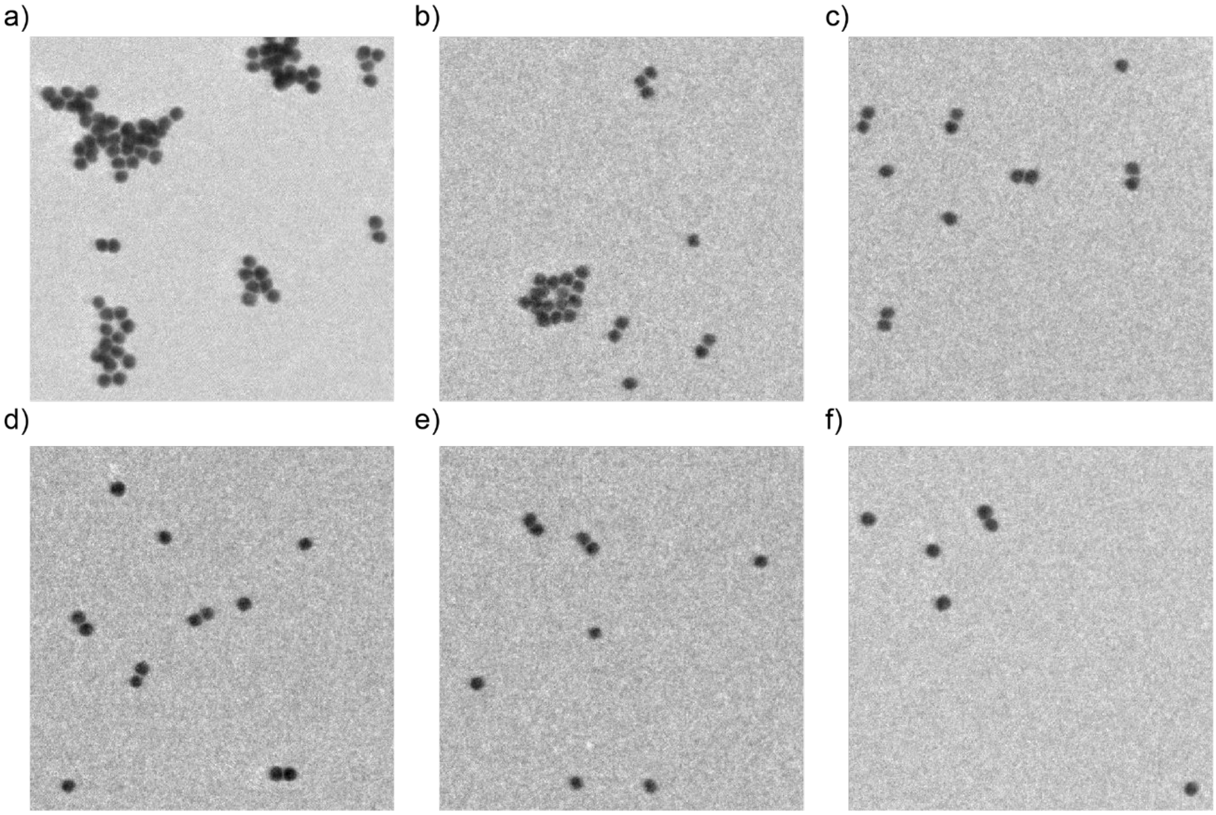

Figure S11. Representative TEM images of DNA-AuNP conjugates of different batches prepared with (a)50% dA-P10, (b) 25% dA-P10, (c)12.5% dA-P10, (d) 6.25% dA-P10, (e) 3.13% dA-P10 and (f) 1.56% dA-P10. The existence of the dimers in each panel and the similar trend as Figure S10 suggest the robustness of this freeing method.

Reference

(1) Ferrari, A. C.; Meyer, J. C.; Scardaci, V.; Casiraghi, C.; Lazzeri, M.; Mauri, F.; Piscanec, S.; Jiang, D.; Novoselov, K. S.; Roth, S.; et al. Raman Spectrum of Graphene and Graphene Layers. *Phys. Rev. Lett.* **2006**, *97* (18), 187401. DOI: 10.1103/PhysRevLett.97.187401.

(2) Ye, Y.; Liu, Z.; Zhang, W.; Cheng, X.; He, S. Freeze-Facilitated Ligand Binding to Plasmonic Gold Nanorods. *Advanced Materials Interfaces* **2019**, *6* (23), 1900975. DOI: 10.1002/admi.201900975.
